# Effects of the monoamine oxidase inhibitors pargyline and tranylcypromine on cellular proliferation in human prostate cancer cells

**DOI:** 10.3892/or.2013.2635

**Published:** 2013-07-24

**Authors:** HYUNG TAE LEE, MI RAN CHOI, MI SOOK DOH, KYOUNG HWA JUNG, YOUNG GYU CHAI

**Affiliations:** Department of Molecular and Life Sciences, Hanyang University, Ansan 426-791, Republic of Korea

**Keywords:** apoptosis, cell cycle, cellular proliferation, pargyline, prostate cancer cells

## Abstract

Chemotherapy is one of the therapeutic strategies that has been used for the inhibition of cancer cell proliferation in several types of cancer, including prostate cancer. Although monoamine oxidase (MAO) inhibitors, phytoestrogen and antioxidants used in chemotherapy have been systematically studied, their effects on cancer cell growth remain to be fully understood. The purpose of this study was to investigate the effects of the MAO inhibitors, pargyline and tranylcypromine on cell survival in human prostate carcinoma (LNCaP-LN3) cells. After treating LNCaP-LN3 cells with pargyline or tranylcypromine, we examined cell proliferation, cell cycle pattern, apoptosis and the expression levels of apoptosis-related genes. The proliferation of cells exposed to pargyline decreased in a dose- and time-dependent manner, while tranylcypromine-treated cells showed the opposite results. Treatment with pargyline significantly induced cell cycle arrest at the G1 phase compared to the control and tranylcypromine-treated cells. In addition, pargyline induced an increase in the cell death rate by promoting apoptosis; however, tranylcypromine had no effect on LNCaP-LN3 cells. Based on our results, we suggest that pargyline is more powerful than tranylcypromine for the treatment of human prostate cancer.

## Introduction

Prostate cancer is the most common non-cutaneous malignancy in Western countries and a major fatal disease in men (1). In particular, prostate cancer-related mortality usually appears in men of advanced age (2). In several different therapeutic strategies for cancer cells, the key focus is the inhibition of cellular proliferation or induction of apoptosis. Previous studies have discovered a number of materials including natural products (such as betulinic acid) and chemical compounds (such as polyamine analogues and everolimus and docetaxel) to prevent the proliferation of cancer cells, and the substances have proven to be effective (3–7).

Recently, various methods including surgery, radiation, chemotherapy and hormonal therapy have been used to treat prostate cancer cells, but, among them, chemopreventive methods are considered key in decreasing progression, mortality, and invasive intervention (8). In chemopreventive methods, administration of phytoestrogen, antioxidant, and cyclooxygenase-2 (Cox-2) selective inhibitors are represented in prostate cancer therapy (2). An alternative to prevent the growth of prostate cancer cells, a selective combination of dietary phytoestrogens (such as genistein, quercetin and biochanin A), was reported to inhibit cell proliferation of androgen-responsive prostate cancer cells (9). Antioxidants, which protect cells from damage caused by oxidative stress, are associated with pathological conditions including inflammation that are a precursor in neoplastic transformation of the prostate (8). Huang *et al*(10) reported that benzodithiazolium-based compound CX9051 is a selective inhibitor for Cox-2 activity, which inhibits cell proliferation and induces apoptosis in numerous human cancer cell types including prostate cancer cells. In addition to these results, various strategies have been extensively studied for prostate cancer therapy.

Previous studies have reported that various monoamine oxidase (MAO) inhibitors including pargyline, tranylcypromine, clorgyline and other derivatives are used for cancer treatment in human cancer cells (11–16). Tranylcypromine, clorgyline and pargyline effectively decreased cell proliferation in various breast cancer cell lines such as MDA-MB-231, MDA-MB-453, MCF-7 and T47D (11,16). Also, in neuroblastoma cells, tranylcypromine showed similar results with breast cancer cells (12). Cortez *et al*(13) reported that after human breast cancer cells were injected into nude mice, regular treatment of pargyline exhibited suppression of tumor growth. Based on these previous studies (11–16), MAO inhibitors may have potential as anticancer agents.

In order to verify the anticancer potential of pargyline and tranylcypromine, we examined the effect of pargyline and tranylcypromine on the cell viability of human prostate carcinoma LNCaP-LN3 cells. After exposing LNCaP-LN3 cells to pargyline or tranylcypromine, we investigated the cell proliferation rate, the cell cycle distribution and the induction of apoptosis in the cells. Furthermore, we analyzed the expression of apoptosis-related genes by treatment of pargyline or tranylcypromine in LNCaP-LN3 cells.

## Materials and methods

### Cell culture

Human prostate carcinoma cells (LNCaP-LN3; KCLB No. 80018) were obtained from the Korean Cell Line Bank (Seoul, Korea). LNCaP-LN3 cells were grown in MEM, supplemented with 10% fetal bovine serum, penicillin (100 U/ml)/streptomycin (100 μg/ml) (all from Invitrogen Life Technologies, Carlsbad, CA, USA) at 37°C in a 5% CO_2_ atmosphere.

### Cell proliferation assay

The proliferation of LNCaP-LN3 cells was evaluated using a Premix WST-1 Cell Proliferation Assay System (Takara Bio, Inc., Shinga, Japan). After exposing the cells to pargyline or tranylcypromine (0.5, 1, 1.5 or 2 mM) for 24, 48, 72, 96 or 120 h, the culture medium was removed and the cells were washed with phosphate buffered saline (PBS). WST-1 reagent was then added, and the cells were incubated for 4 h. The results of the WST-1 assay were measured using a Model 680 microplate reader (Bio-Rad, Hercules, CA, USA).

### Cell cycle analysis

The cell cycle assay was performed as previously described (17). The cells were plated in 10-cm^2^ plates (Corning Inc., Corning, NY, USA) and cultured in normal growth medium for 24 h before treatment with pargyline or tranylcypromine (Sigma-Aldrich, St. Louis, MO, USA). After treating with pargyline or tranylcypromine for 24 or 48 h, the cells were harvested to analyze cell cycle using 0.25% trypsin-EDTA (Invitrogen Life Technologies). The cells were washed twice with PBS, and probed with BD CycleTest™ Plus DNA Reagent kit (BD Biosciences, Franklin Lakes, NJ, USA) according to the manufacturer’s instructions. Cell cycle distribution was analyzed using FACSCalibur (BD Biosciences). The percentage of cells in different cell cycle phases was calculated using ModFit LT 3.0 (Verity Software House, Topsham, ME, USA).

### Real-time RT-PCR

After exposing LNCaP-LN3 cells to pargyline or tranylcypromine, total RNA was isolated using RNAiso Plus (Takara Bio, Inc.). Total RNA was reverse transcribed into cDNA using PrimeScript™ Reverse Transcriptase (Takara Bio, Inc.). Real-time PCR was performed using 7500 real-time PCR system (Applied Biosystems, Foster City, CA, USA) and 2X SYBR-Green PCR Master Mix (Takara Bio, Inc.). The sequences of the primers used in this study were: *BCL-2* forward, 5′-GGGGAGGATTGTGGCCTTC-3′ and reverse, 5′-CAGGGCGATGTTGTCCACC-3′; *NOXA* forward, 5′-ACCAAGCCGGATTTGCGATT-3′ and reverse, 5′-ACTTGCACTTGTTCCTCGTGG-3′; and *β-actin* forward, 5′-TGGAGAAAATCTGGCACCACACC-3′ and reverse, 5′-GATGGGCACAGTGTGGGTGACCC-3′. β-actin was used as an internal standard. The gene expression levels were analyzed using the 2^−ΔΔCT^ method (18).

### Apoptosis analysis

Cells were plated at 1×10^6^ cells/cm^2^ in 10-cm^2^ plates and grown for 24 h before treatment with pargyline or tranylcypromine. After treating with pargyline or tranylcypromine for 24 h, the cells were harvested with 0.25% trypsin-EDTA and were washed twice with PBS. The apoptosis analysis was performed using *In Situ* Cell Death Detection kit, Fluorescein (Roche Diagnostics, Mannheim, Germany), according to the manufacturer’s instructions, and analyzed using a FACSCalibur (BD Biosciences).

### Western blot analysis

Western blotting was performed as previously described (19), with minor modifications. After treating the cells with 0.5 mM pargyline or tranylcyprominein for 24 h, extraction of total protein from the cells was performed using RIPA buffer [50 mM Tris-HCl, pH 7.5; 150 mM NaCl; 1% (v/v) Nonidet P-40 (NP-40); 0.5% sodium deoxycholate; 0.1% SDS and protease inhibitors]. The protein was separated by SDS-PAGE and transferred to polyvinylidene difluoride membranes (Schleicher & Schuell BioScience Inc., Keene, NH, USA). The membranes were incubated overnight at 4°C with a BCL-2 antibody, cytochrome *c* antibody (both from Santa Cruz Biotechnology, Inc., Santa Cruz, CA, USA), caspase-3 antibody (Cell Signaling Technology, Inc., Danvers, MA, USA) or β-actin antibody (Sigma-Aldrich) followed by incubation with HRP-conjugated anti-rabbit or anti-mouse IgG. After washing with TBS-T, the proteins were visualized with ECL^TM^ Western Blotting Detection Reagents (GE Healthcare, Wauwatosa, WI, USA).

### Statistical analyses

The data were analyzed using OriginPro 8 software (OriginLab Corp., Northampton, MA, USA). Each value is expressed as the means ± standard error of mean (SEM) from 3 independent experiments. All statistical analyses were performed using SPSS 17.0 software (SPSS Inc., Chicago, IL, USA). P-values <0.05 were considered to indicate statistically significant differences.

## Results

### Regulation of cell proliferation by pargyline and tranylcypromine

**Figure 1:**
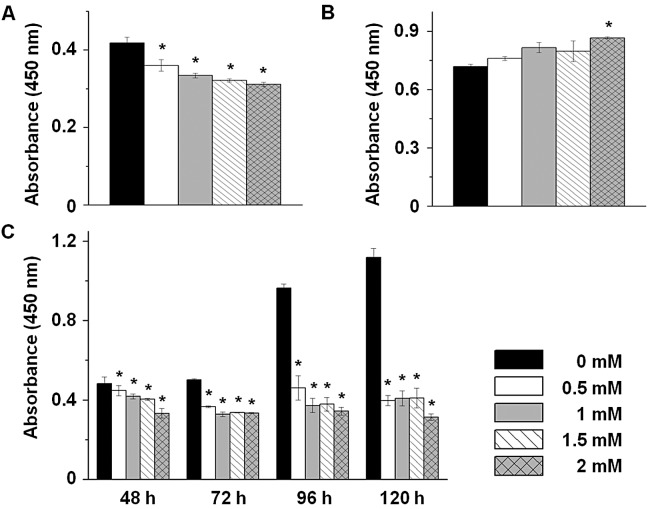
The effect of pargyline and tranylcypromine in the cell proliferation of human prostate cancer cells. LNCaP-LN3 cells were exposed to pargyline or tranylcypromine in a dose-dependent manner (0, 0.5, 1, 1.5 and 2 mM). After the treatment, the cell proliferation was measured by WST-1 assay. (A) LNCaP-LN3 cells exposed to pargyline for 24 h. (B) LNCaP-LN3 cells exposed to tranylcypromine for 24 h. (C) Cells exposed to pargyline for 48, 72, 96 and 120 h. The values represent the means ± SE (n=3). ^*^Significantly different from 0 mM. For statistical analyses, we conducted a one-way ANOVA followed by Tukey’s HSD post-hoc test.

To investigate the cellular proliferation effect of MAO inhibitors on prostate cancer cells, we performed a cell proliferation assay in LNCaP-LN3 cells after exposing the cells to pargyline or tranylcypromine treatment in a dose-dependent manner (0, 0.5, 1, 1.5 and 2 mM) for 24 h. The cells exposed to pargyline exhibited a decrease in cellular proliferation (Fig. 1A) that was dose-dependent. By contrast, the cells exposed to tranylcypromine exhibited an increase in cellular proliferation compared to the control cells (Fig. 1B). To further investigate the effect of pargyline in a time-dependent manner, we exposed the cells to pargyline for 48, 72, 96 and 120 h. The proliferation in the control cells increased continuously, while the proliferation in the cells exposed to pargyline did not increase and, markedly, the cells exposed to 2 mM pargyline for 120 h decreased 3-fold in cellular proliferation compared to the control cells (Fig. 1C). Therefore, pargyline may inhibit the proliferation of prostate cancer cells in a time- and dose-dependent manner.

### Regulation of cell cycle patterns by pargyline and tranylcypromine

**Figure 2:**
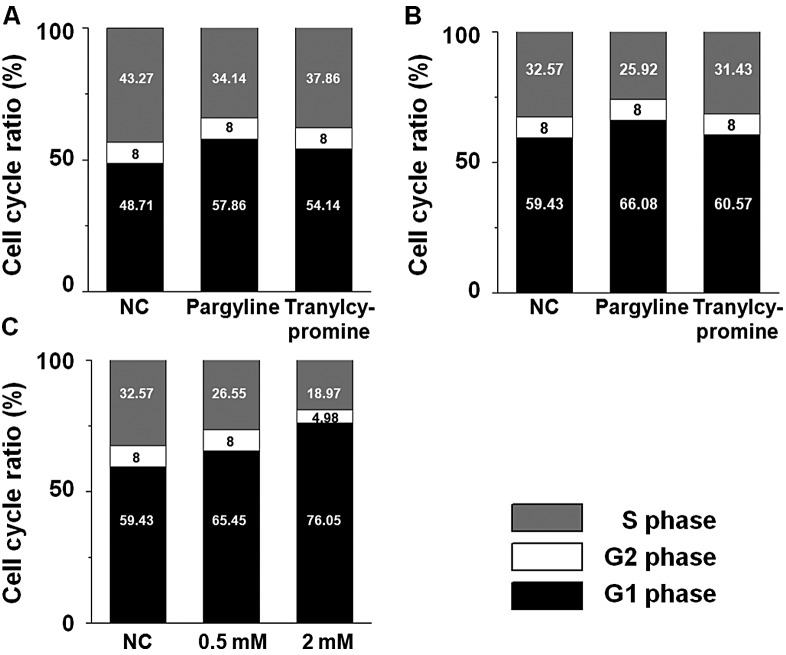
The alteration of the cell cycle of human prostate cancer cells exposed by pargyline or tranylcypromine. LNCaP-LN3 cells were exposed to pargyline or tranylcypromine in a time- and dose-dependent manner and cell cycle was analyzed using flow cytometry. (A and B) The cells were exposed to pargyline or tranylcypromine for 24 and 48 h, respectively. (C) The cells were exposed to 0.5 and 2 mM pargyline for 24 h.

Based on these observations that pargyline and tranylcypromine affect the cellular proliferation in prostate cancer cells, we examined whether the proliferation changes in the cells exposed to pargyline or tranylcypromine were induced by alteration of the cell cycle pattern. The S phase ratio of the cells exposed to pargyline for 24 and 48 h decreased, while their G1 phase ratio increased compared to the control cells (Fig. 2A and B). In particular, the decrease in the S phase or the increase in the G1 phase became more evident with the progress of time. On the other hand, there was little difference between the control and the tranylcypromine-exposed cells at the alteration ratios of the S and the G1 phase. We further analyzed whether pargyline affected the cell cycle pattern in a dose-dependent manner. When the cells were exposed to 0.5 or 2 mM pargyline for 24 h, the decrease of S phase and the increase of the G1 phase in the cells were dose-dependent compared to the control cells (Fig. 2C).

### Regulation of apoptosis-related genes by pargyline and tranylcypromine

**Figure 3:**
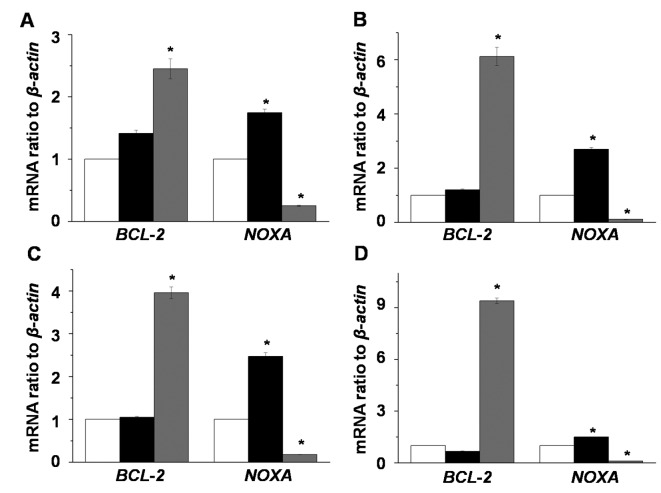
The expression of apoptosis-related genes altered in human prostate cancer cells exposed to pargyline and tranylcypromine. LNCaP-LN3 cells were exposed to 2 mM pargyline or tranylcypromine in a time-dependent manner. The expression pattern of apoptosis-related genes such as *BCL-2* (anti-apoptotic protein) and *NOXA* (pro-apoptotic protein) was analyzed using real-time RT-PCR. (A–D) The cells were exposed to pargyline or tranylcypromine for 6, 12, 24 and 48 h, respectively. White, untreated cells (NC); black, pargyline-treated cells; gray, tranylcypromine-treated cells. The values represent the means ± SEM (n=3). ^*^Significantly different from NC cells. For statistical analyses, we conducted a one-way ANOVA followed by Tukey’s HSD post-hoc test.

To investigate the induction of apoptosis by MAO inhibitors in LNCaP-LN3 cells, we analyzed the expression changes of apoptosis-related genes (*BCL-2* and *NOXA*) after exposing LNCaP-LN3 cells to 2 mM pargyline or tranylcypromine in a time-dependent manner (Fig. 3). The expression level of *BCL-2* mRNA did not change in the pargyline-treated cells, while its expression levels in the tranylcypromine-treated cells were significantly increased compared to the control and pargyline-treated cells. On the other hand, the expression level of *NOXA* mRNA in the pargyline-treated cells significantly increased, while its expression in tranylcypromine-treated cells decreased.

### Induction of apoptosis by pargyline and tranylcypromine

**Figure 4:**
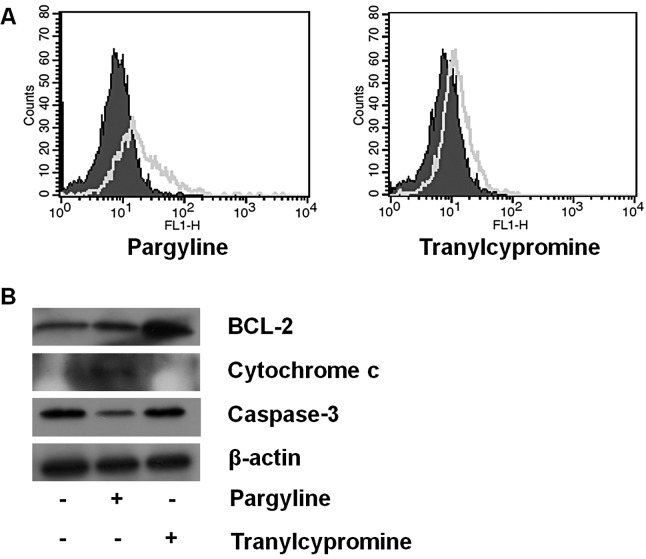
Effect of pargyline and tranylcypromine on the induction of apoptosis in human prostate cancer cells. (A) After exposing LNCaP-LN3 cells to 0.5 mM pargyline or tranylcypromine for 24 h, the cells were analyzed for the rate of cell death using *in situ* assay. (B) After exposing LNCaP-LN3 cells to 2 mM pargyline or tranylcypromine for 48 h, the cells were analyzed for the expression of apoptosis regulatory proteins (BCL-2, cytochrome *c* and caspase-3) using western blotting.

Based on the results described above, we observed the apoptotic signals after exposing the cells to pargyline or tranylcypromine for 24 h. The pargyline-treated cells showed more apoptotic induction, whereas the tranylcypromine-treated cells showed a similar pattern compared to the control cells (Fig. 4A). In addition to the increase in the apoptotic cells after pargyline treatment, we further examined the expression level of the apoptosis regulatory proteins (BCL-2, cytochrome *c* and caspase-3) after pargyline or tranylcypromine treatment for 48 h (Fig. 4B). Pargyline treatment induced an increase of cytochrome *c* and a decrease of caspase-3 in the cells, but did not lead to a change of BCL-2 expression. On the other hand, tranylcypromine promoted a significant increase of BCL-2, showing no change of cytochrome *c* and caspase-3 expressions. Therefore, pargyline may induce cell death in prostate cancer cells.

## Discussion

Prostate cancer is the most common malignancy in Western countries, particularly in >50-year-old men (1,2). Prostate cancer-related mortality was reported as the second most common among all types of cancer (20). Treatments such as chemopreventive methods have been systematically studied for prostate cancer therapy (8). Additionally, various chemical reagents are being used to prevent cancer growth due to the importance of inhibition of proliferation in cancer cells. However, there are currently no effective target materials to prevent the growth of the cancer cells. For this reason, we analyzed the possibility of using pargyline and tranylcypromine as candidates for the treatment of prostate cancer.

In recent years, MAO inhibitors such as pargyline, tranylcypromine and clorgyline (11,12,14–16,21) began to be tested for cancer cell treatment. In the present study, we found that pargyline efficiently inhibited the proliferation of prostate cancer cells. In a study reported by Flamand *et al (*14), clorgyline suppressed the enzyme activity of MAO A as well as the cellular proliferation in prostate cancer cells. A previous study observed that pargyline, tranylcypromine and clorgyline significantly inhibited the growth of neuroblastoma cells in a concentration-dependent manner (15). In addition, the MAO inhibitors are known to suppress the growth of breast cancer cell lines such as MDA-MB-231, MDA-MB-453, MCF-7 and T47D (11,16,21). Therefore, it may be that pargyline inhibits the proliferation of various cancer cells including prostate and breast cancer cells. On the other hand, Benelkebir *et al*(22) reported that tranylcypromine analogues suppressed cell growth more effectively than tranylcypromine in prostate cancer cells. In the present study, tranylcypromine did not inhibit the cell proliferation in prostate cancer cells. Therefore, it is believed that tranylcypromine differentially affects cell proliferation according to cancer type whereas pargyline inhibits cellular proliferation in cancer cells regardless of cancer type.

It was reported that cell cycle arrest at the G1 phase negatively affects cell proliferation (23). Our data showed a decrease in the S phase proportion and an increase in the G1 phase proportion by pargyline treatment in prostate cancer cells. In particular, pargyline led to a decrease in the S phase proportion and an increase in the G1 phase proportion in a dose-dependent manner. A previous study reported that pargyline induced cell cycle arrest by the decrease of cyclin B1 protein in human cervical adenocarcinoma HeLa cells (24). In the present study, tranylcypromine, unlike pargyline, did not show a significant regulation in the proportion of S phase compared to the control. Contrary to our result, Gatta and Mantovani (25) observed that tranylcypromine treatment (2 μM) in human colon carcinoma HCT116 cells efficiently suppressed progression to the S phase. Based on the previous report and our results, tranylcypromine may show a different effect depending on the concentration of tranylcypromine and type of cancer cells. Therefore, these results suggest that pargyline is more powerful to induce cell cycle arrest in prostate cancer cells than tranylcypromine.

**Figure 5:**
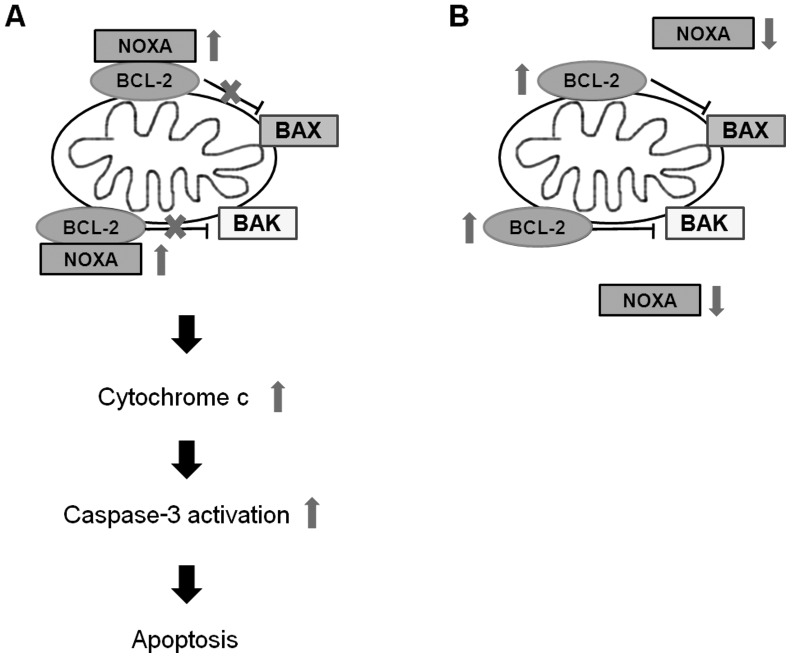
Schematic diagram of the pargyline and tranylcypromine effects on apoptotic pathway in prostate cancer cells.

We observed that the expression of apoptosis related-genes such as *BCL-2* and *NOXA* was regulated by pargyline or tranylcypromine. BCL-2 is an anti-apoptotic protein that regulates apoptosis by inhibition of the release of pro-apoptotic factors such as cytochrome *c*(26–28). NOXA, BH3-only protein, is a pro-apoptotic member of the BCL-2 protein family, and interacts with MCL1, BCL-2 like 1 and BCL-2 (29). In the present study, the expression levels of mRNA and protein of *BCL-2* were increased only by tranylcypromine treatment, while the mRNA expression of *NOXA* exhibited an increase by pargyline but a decrease by tranylcypromine. Previous studies reported that NOXA BH3 domain interacts with BCL-2 BH3 binding groove, and it indirectly promotes mitochondrial dysfunction through BAX and BAK by inhibiting anti-apoptotic protein BCL-2 (29–32). BCL-2 is known to prevent the release of cytochrome *c* from mitochondria, which activates caspase-9, followed by caspase-3 cleavage in the intrinsic pathway of apoptotic events (33,34). We identified that pargyline led to an increase of cytochrome *c* and a decrease of caspase-3 in prostate cancer cells. Therefore, pargyline may promote apoptosis through regulation of BCL-2 and NOXA expression, while tranylcypromine does not affect apoptosis in our condition. As an alternative, we confirmed that pargyline effectively induced apoptosis compared with tranylcypromine treatment in prostate cancer cells using an *in situ* cell death detection technique. Similar to our results, Malorni *et al*(35) reported that apoptosis in human melanoma M14 cells exposed to 1 mM pargyline increased. In addition, it has been reported that the periodic administration of pargyline into nude mouse models significantly induced apoptosis of tumors formed by breast cancer MCF-7 cells (13). On the other hand, tranylcypromine treatment, in combination with HDAC inhibitors, induced synergistic apoptotic cell death in glioblastoma multiforme cells (36). Collectively, the differential expression of BCL-2 and NOXA may cause the opposite effects of pargyline and tranylcypromine to cellular proliferation in prostate cancer cells (Fig. 5).

In summary, we observed the mechanisms associated with cell proliferation and apoptosis in prostate cancer cells after exposing the cells to the MAO inhibitors, pargyline and tranylcypromine. Pargyline not only induced growth arrest in the cells but also showed a more significant increase of the G1 phase and a decrease of the S phase compared with tranylcypromine. Furthermore, when pargyline was used to treat prostate cancer cells, the expression of pro-apoptotic member NOXA increased significantly, but the expression of anti-apoptotic protein BCL-2 decreased compared with tranylcypromine treatment. The increased expression of cytochrome *c* by pargyline treatment induces a decrease in the expression of caspase-3 under pargyline-exposed conditions. In addition, the treatment of pargyline effectively induces apoptosis more than the treatment of tranylcypromine. Based on our results and previous studies, it is believed that pargyline has greater pharmaceutical potential as an anticancer drug than tranylcypromine for human prostate cancer.
